# Retention enema with traditional Chinese medicine for hepatic encephalopathy: A protocol for a systematic review and meta-analysis

**DOI:** 10.1097/MD.0000000000022517

**Published:** 2020-10-02

**Authors:** Xiao Liang, Lihong Wen, Yifang Wu, Yanmin Hao, Shaobo Wang, Xiaoyu Hu

**Affiliations:** a Department of Infectious Diseases, Hospital of Chengdu University of Traditional Chinese Medicine; b National Integrative Medicine Clinical Base for Infectious Diseases, Chengdu 610072; c Emergency Department, Hospital of Chengdu University of Traditional Chinese Medicine, Chengdu 610072; d Intensive Care Unit, Anyang District Hospital, Anyang 455000, PR China.

**Keywords:** hepatic encephalopathy, meta-analysis, protocol, retention enema, traditional Chinese medicine

## Abstract

**Background:**

Hepatic encephalopathy (HE) is one of the common complications of many serious liver diseases. Western medicine treatment is mainly symptomatic treatment such as neutralizing blood ammonia and protecting liver, which has poor curative effect, easy repetition and high mortality. Retention enema with traditional Chinese medicine (TCM) has been used on treatment of HE in China for many years. And it has been clinically proved that retention enema with TCM is effective and safe. But there is absent convincing evidence-based medicine to confirm the efficacy of retention enema with TCM for HE. Thus, we aimed to conduct this meta-analysis to summarize the efficacy of retention enema with TCM in patients with HE.

**Methods:**

The study only selects clinical randomized controlled trials of retention enema with TCM for HE. We will search each database from the built-in until December 31, 2020. The English literature mainly searches Cochrane Library, Pubmed, EMBASE, and Web of Science. While the Chinese literature comes from CNKI, CBM, VIP, and Wanfang database. Meanwhile, we will retrieve clinical trial registries and gray literature. Two researchers worked independently on literature selection, data extraction and quality assessment. The dichotomous data is represented by relative risk (RR), and the continuous is expressed by mean difference (MD) or standard mean difference (SMD), eventually the data is synthesized using a fixed effect model (FEM) or a random effect model (REM) depending on the heterogeneity. The total effective rate, blood ammonia and the total bilirubin were evaluated as the main outcomes. While several secondary outcomes were also evaluated in this study. The statistical analysis of this Meta-analysis was conducted by RevMan software version 5.3.

**Results:**

This study will synthesize and provide high-quality evidence based on the data of the currently published retention enema with TCM for the treatment of HE.

**Conclusion:**

This meta-analysis aims to evaluate the benefits of retention enema with TCM for the treatment of HE reported in randomized controlled trials, and provide more options for clinicians and patients with HE.

**Registration number:**

INPLASY202080107.

## 1 Introduction

Hepatic encephalopathy(HE) is a syndrome caused by severe liver diseases, which is based on metabolic disorders and characterized by central nervous system dysfunction.[^1^,^2^] Its main clinical manifestations are disturbance of consciousness, behavioral disorders, and coma.^[3]^ The incidence of hepatic encephalopathy is high, and it is one of the common complications of liver diseases such as severe hepatitis, cirrhosis, and liver cancer.[^1^,^4^] The mechanism of hepatic encephalopathy is complex, it is currently believed that the pathogenesis of this disease is closely related to the following mechanisms: ammonia poisoning theory, inflammatory reaction injury theory, amino acid imbalance theory and pseudo neurotransmitter theory, gamma aminobutyric acid/benzodiazepine compound receptor hypothesis, manganese poisoning theory, brain stem reticular system dysfunction.[^5^,^6^,^7^,^8^,^9^,^10^,^11^] Among them, the theory of ammonia poisoning is recognized by the academic circles.^[12]^ It is generally believed that when the liver function is seriously damaged, the livers ability to synthesize urea and remove ammonia is reduced. Many toxic metabolites from the intestinal tract are not detoxified and cleared by the liver. They enter the systemic circulation through the portal collateral circulation, and then pass through the blood-brain barrier to the brain, causing brain dysfunction.

Western medicine treatment of hepatic encephalopathy is mainly to neutralize blood ammonia, protect liver function, supplement electrolytes, diuresis, and other symptomatic treatment, but the treatment does not cure the root cause, easy recurrence, and high mortality.[^13^,^14^,^15^,^16^] In addition, such patients often have mental changes, do not cooperate in the treatment process, oral administration is difficult. Therefore, non-oral administration of traditional Chinese medicine retention enema technology may become a new treatment method for hepatic encephalopathy.

The technology of retention enema with traditional Chinese medicine has a long history and is one of the commonly used treatment methods in traditional Chinese medicine.[^17^,^18^,^19^,^20^] On the basis of syndrome differentiation and treatment of traditional Chinese medicine, drug retention enema is used to reduce blood ammonia. This method has attracted wide attention of clinical workers in the treatment of hepatic encephalopathy. Thus, we intend to collect randomized controlled trials (RCTs) about retention enema with TCM for HE based on evidence-based medicine, and conduct a meta-analysis of its efficacy to provide higher quality clinical evidence for retention enema with TCM is beneficial for patients with hepatic encephalopathy.

## 2 Methods

### 2.1 Protocol registration

The systematic review protocol has been registered on the INPLASY website (https://inplasy.com/inplasy-2020-8-0107/) and INPLASY registration number is INPLASY202080107. It is reported following the guidelines of Cochrane Handbook for Systematic Reviews of Interventions and the Preferred Reporting Items for Systematic Reviews and Meta-analysis Protocol (PRISM).^[21]^ If there are any adjustments throughout the study, we will fix and update the details in the final report.

### 2.2 Inclusion criteria

#### 2.2.1 Study design

The study only selects clinical RCTs of retention enema with TCM for HE published in both Chinese and English. However, animal experiments, reviews, case reports, and non-randomized controlled trials are excluded.

#### 2.2.2 Participants

The patients with clinically diagnosed HE and treatment with herb retention-enema, regardless of race, gender, and age. Patients with severe heart disease, liver and kidney dysfunction, mental illness, or a relevant drug allergic history will be not included.

#### 2.2.3 Interventions

Both groups were given routine comprehensive medical treatment. On the basis of these treatments, the experiment group used retention enema with TCM, while the control group applied for placebo, or no treatment. In addition, the 2 groups did not take any drugs that interfered with the outcome indicators.

#### 2.2.4 Outcomes

The primary outcomes include the total effective rate, blood ammonia, and the total bilirubin (TBIL).

The secondary outcomes include the awake time (time from the beginning of treatment to waking up), alanine transaminase (ALT), aspartate transaminase (AST), γ-glutamyl transpeptidase (GGT), albumin.

### 2.3 Search methods

#### 2.3.1 Electronic searches

Information sources: We will retrieve each database from the built-in until December 31, 2020. The English literature mainly searches Cochrane Library, Pubmed, EMBASE, and Web of Science. While the Chinese literature comes from CNKI, Wanfang CBM, and VIP database. We adopt the combination of heading terms and free words as search strategy which decided by all the reviewers. Search terms: retention enema with traditional Chinese medicine, retention enema with Chinese medicine, retention enema with TCM, Chinese medicine enema, Chinese medicine retention enema, herbal retention enema, herbal enema, herb retention-enema, retention enema, enema, hepatic encephalopathy, HE, portal-systemic encephalopathy, hepatocerebral encephalopathy, portosystemic encephalopathy, hepatic coma, hepatic comas, hepatic stupors, fulminant hepatic failure with cerebral edema. We will simply present the search process of the Cochrane library, as shown in Table 1, adjusting different search methods according to different Chinese and English databases.

**Table 1:**
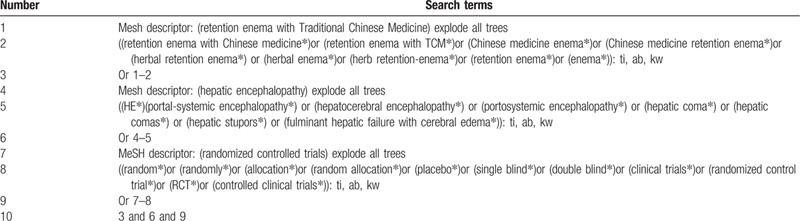
Example of Cochrane search strategy.

#### 2.3.2 Searching other resources

At the same time, we will retrieve other resources to complete the deficiencies of the electronic databases, mainly searching for the clinical trial registries and gray literature about retention enema with TCM for HE on the corresponding website.

### 2.4 Data collection and analysis

#### 2.4.1 Selection of studies

**Figure 1:**
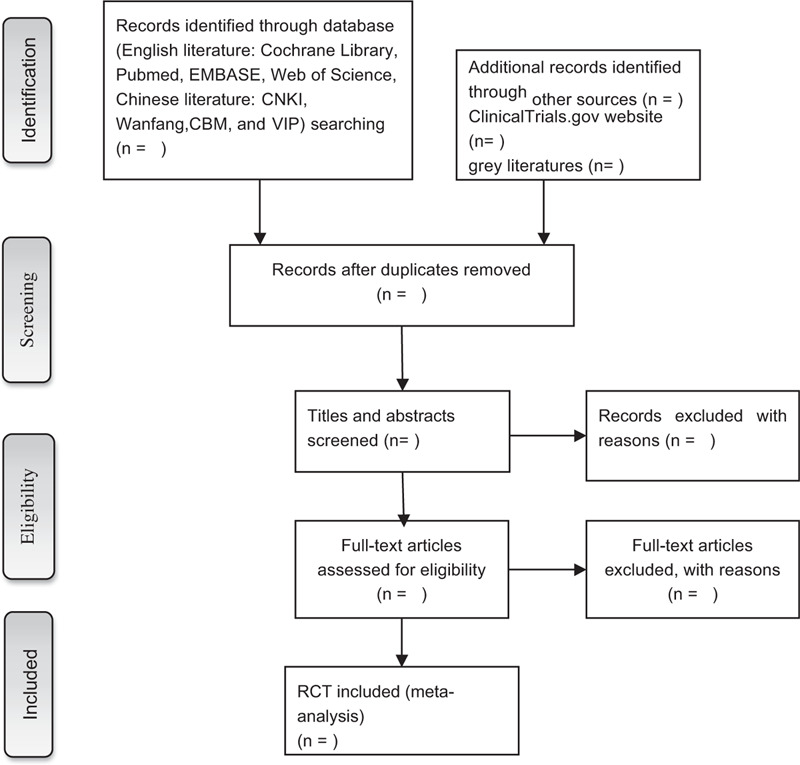
Flow chart of the study selection.

Import all literatures that meet the requirements into Endnote X8 software. Firstly, 2 independent reviewers initially screened the literatures that did not meet the pre-established standards of the study by reading the title and abstract. Secondly, download the remaining literatures and read the full text carefully to further decide whether to include or not. Finally, the results were cross-checked repeatedly by reviewers. If there is a disagreement in the above process, we can reach an agreement by discussing between both reviewers or seek an opinion from third party. PRISMA flow diagram (Fig. 1) will be used to show the screening process of the study.

#### 2.4.2 Data extraction and management

According to the characteristics of the study, we prepare an excel form for data collection before data extraction. Outcome indicators for eligible studies were independently extracted and filled in the data extraction form by 2 reviewers. If there is any argument, it can get an agreement by discussing through 2 reviewers or seek suggestions form third party. The main data extracted are as follows: title, author, year, sample size, duration of disease, interventions, outcome measures, adverse reactions, follow-up, etc. If we find something unclear in the study, we can contact the author of the communication directly for more detailed information. The above information was finally cross-checked by 2 reviewers.

#### 2.4.3 Assessment of risk of bias in included studies

The quality assessment of RCTs adopts the risk of bias (ROB) assessment tool provided by the *Cochrane Handbook*. The following 7 items, such as random sequence generation, allocation concealment, blinding of participants and personnel, blinding of outcome assessment, incomplete outcome data, selective outcome reporting, and other bias, are evaluated by 3 grades of “low bias”, “high bias”, and “unclear bias”. The discrepancies will get a consistent conclusion by discussing between both reviewers or seeking the third-party consultation.

#### 2.4.4 Measures of treatment effect

Different evaluation methods are selected according to the different efficacy indicators. For the dichotomous data, we will choose the effect scale indicator relative risk (RR) with 95% confidence interval (CI) to represent. While the continuous data is expressed as mean difference (MD) or standardized mean difference (SMD) with 95% CI depending on whether the measurement scale is consistent or not.

#### 2.4.5 Dealing with missing data

The reviewers will contact the first author or correspondent author via email or telephone to obtain missing data if the relevant data is incomplete. If the missing data is still not obtained in the above way, we can synthesize the available data in the initial analysis. Furthermore, sensitivity analysis will be used to assess the potential impact of missing data on the overall results of the study.

#### 2.4.6 Assessment of heterogeneity

Heterogeneity will be assessed by Chi-Squared test and *I* ^2^ test. If *I* ^2^ < 50%, *P* > .1, we consider that no statistical heterogeneity between each study and choose fixed effect model (FEM) to synthesize the data. If *I* ^2^ ≥ 50%, *P* < .1, indicating that there is a statistical heterogeneity, the data is integrated by the random effect model (REM). In addition, due to differences in heterogeneity, we will conduct subgroup or sensitivity analysis to look for the potential causes.

#### 2.4.7 Data analysis

Review Manager software version 5.3 provided by the Cochrane Collaboration will be performed for data synthesis and analysis. The dichotomous data is represented by RR, continuous data is expressed by MD or SMD. If there is no heterogeneity (*I* ^2^ < 50%, *P* > .1), the data is synthesized using a fixed effect model. Otherwise (*I* ^2^ ≥ 50%, *P* < .1), a random effect model is used to analyze. Then subgroup analysis will be conducted based on the different causes of heterogeneity. If a meta-analysis cannot be performed, it will be replaced by a general descriptive analysis.

#### 2.4.8 Subgroup analysis

If the results of the study are heterogeneous, we will conduct a subgroup analysis for different reasons. Heterogeneity is manifested in the following several aspects, such as race, age, gender, different intervention forms, pharmaceutical dosage, treatment course.

#### 2.4.9 Sensitivity analysis

Sensitivity analysis is mainly used to evaluate the robustness of the primary outcome measures. The method is that removing the low-level quality study one by one and then merging the data to assess the impact of sample size, study quality, statistical method, and missing data on results of meta-analysis.

#### 2.4.10 Grading the quality of evidence

In this systematic review, the quality of evidence for the entire study is assessed using the “Grades of Recommendations Assessment, Development and Evaluation (GRADE)” standard established by the World Health Organization and international organizations.^[22]^ To achieve transparency and simplification, the GRADE system divides the quality of evidence into 4 levels: high, medium, low, and very low.

## 3 Discussion

Retention enema with TCM is based on the theory of syndrome differentiation and treatment. It can reduce blood ammonia level by herbal enema, the commonly used Chinese herbal medicines are rhubarb, radix paeoniae rubra, borneol, and Gardenia jasminoides. This treatment has attracted more and more attention of clinical workers in the treatment of HE. This study reviewed the RCTs of herbal retention enema combined with conventional western medicine in the treatment of HE and conducted a meta-analysis to evaluate the clinical effect of this treatment on HE. Finally, it provides a strong basis for the treatment of HE with herbal enema.

In this study, we attempt to provide a new option for treatment with HE. We hope this study can provide a comprehensive assessment to whether retention enema with TCM is beneficial for HE. Moreover, RCTs will be included in our studies and appear to be high quality and low risk of bias. However, there may be some limitations in our meta-analysis. Firstly, both Chinese and English forms of research may increase the bias of the study. Secondly, the variety of race, age, gender, intervention forms, pharmaceutical dosage, and treatment course may result in higher clinical and statistical heterogeneity.

In conclusion, we hope this study will provide higher quality evidence for the benefits of retention enema with TCM for patients with HE. Thus, retention enema with TCM has become an alternative method for the treatment of HE.

## Author contributions

**Conceptualization:** Xiao Liang.

**Data curation:** Xiao Liang, Lihong Wen, Yanmin Hao.

**Formal analysis:** Xiao Liang, Lihong Wen, Yifang Wu.

**Funding acquisition:** Xiaoyu Hu.

**Methodology:** Xiao Liang, Lihong Wen, Shaobo Wang.

**Project administration:** Xiao Liang, Yifang Wu.

**Resources:** Yanmin Hao, Shaobo Wang.

**Software:** Xiao Liang, Lihong Wen, Yanmin Hao.

**Supervision:** Xiaoyu Hu.

**Writing – original draft:** Xiao Liang, Lihong Wen.

**Writing – review & editing:** Xiao Liang, Xiaoyu Hu.
